# Dectin-1 Promotes Type I and III Interferon Expression to Support Optimal Antifungal Immunity in the Lung

**DOI:** 10.3389/fcimb.2020.00321

**Published:** 2020-07-08

**Authors:** Orchi Dutta, Vanessa Espinosa, Keyi Wang, Samantha Avina, Amariliz Rivera

**Affiliations:** ^1^Graduate School of Biomedical Sciences, Rutgers Biomedical and Health Sciences, Newark, NJ, United States; ^2^Center for Immunity and Inflammation, Rutgers Biomedical and Health Sciences, Newark, NJ, United States; ^3^Department of Pediatrics, Center for Immunity and Inflammation, Rutgers Biomedical and Health Sciences, Newark, NJ, United States

**Keywords:** *Aspergillus*, dectin-1, β-glucan, cell wall, innate immunity, IFN-λ

## Abstract

Pulmonary infections with *Aspergillus fumigatus* (*Af*) are a significant cause of invasive fungal disease and lead to high morbidity and mortality in diverse populations throughout the world. Currently available antifungal drugs are often ineffective, thus contributing to unacceptably high mortality rates in patients suffering from invasive fungal infections. The use of cytokines as adjunctive immune therapies holds the promise of significantly improving patient outcomes in the future. In recent studies, we identified an essential role for type I and III interferons as regulators of optimal antifungal responses by pulmonary neutrophils during infection with *Af*. Although various membrane and cytosolic nucleic acid sensors are known to regulate interferon production in response to viruses, the pathways that regulate the production of these cytokines during fungal infection remain uncovered. In the current study, we demonstrate that dectin-1-mediated recognition of β-glucan on the cell wall of the clinically relevant fungal pathogen *Aspergillus fumigatus* promotes the activation of a protective cascade of type I and III interferon expression. We further demonstrate that exogenous administration of type I and III interferons can rescue inadequate antifungal responses in dectin-1^−/−^ mice, suggesting the potential therapeutic benefit of these cytokines as activators of antifungal defense in the context of innate defects.

## Introduction

Fungal infections affect close to one billion individuals worldwide, with a range of diseases spanning mild or superficial skin infections to invasive or chronic infections that can become life-threatening (Bongomin et al., 2017). Systemic fungal infections cause severe disease in immunocompromised patients. Fatal fungal infections cause hundreds of thousands of deaths in patients undergoing chemotherapy and account for almost 50% of all HIV/AIDS-related deaths (Denning, 2016; GAFFI, 2017). *Aspergillus fumigatus* (*Af*) is the most common etiological agent of invasive aspergillosis and remains a challenging infection to treat (Brown et al., 2012). The five current antifungal drug classes have limited effectiveness, while accompanied by a plethora of side effects and prohibitive toxicity (Pianalto and Alspaugh, 2016; Mourad and Perfect, 2018). Successful targeting of fungal pathogens has been challenging due to the relative similarities between human and fungal cells (Scorzoni et al., 2017). As a consequence, there is a necessity for developing more effective treatments or combination therapies. In this setting, immunomodulation poses a promising avenue of combination therapeutics or prophylaxis. For example, treatment with cytokines or adoptive transfer of cells that are immunocompetent or pre-activated with *Aspergillus* spores has been shown to assist in controlling *Aspergillus* infection (Bozza et al., 2003; Perruccio et al., 2005; Deo and Gottlieb, 2015; Salazar and Brown, 2018; Sam et al., 2018). However, in order to develop and employ such strategies, we require a deeper understanding of the intricacies of host interactions with *Aspergillus*.

Recognition of *Aspergillus* spores is the earliest host-pathogen event to trigger an immune response. C-type lectin receptors (CLRs) are one class of pattern recognition receptors (PRRs) important for the recognition of carbohydrate pathogen-associated molecular patterns (PAMPs) expressed on the cell wall of *Aspergillus* and other fungi (Gresnigt et al., 2012). Dectin-1 is a CLR expressing a single carbohydrate recognition domain (CRD) and atypical immunoreceptor tyrosine-based activation motif (ITAM) for the activation of downstream signaling (Brown, 2005; Heinsbroek et al., 2006; Schorey and Lawrence, 2008). The ligands for dectin-1 are β-1,3- and β-1,6-linked glucans. These rich carbohydrate residues are found embedded in the cell walls of numerous bacteria and fungi (Brown and Gordon, 2001; Herre et al., 2004; Taylor et al., 2007). Although the core of the *Aspergillus* cell wall consists of β-glucans, in resting conidia, these residues are shielded under layers of melanin and highly hydrophobic rodlets (Gow et al., 2017). Once it differentiates into its filamentous form, the cell wall of *Aspergillus* becomes less organized, expresses other complex polysaccharides, and has limited β-glucan exposure (Hohl et al., 2005; Gravelat et al., 2013; Gow et al., 2017). However, when *Aspergillus* conidia initially begin germinating, β-glucan is available for binding by dectin-1 (Tronchin et al., 1995; Hohl et al., 2005; Steele et al., 2005; Gersuk et al., 2006). The downstream outcomes of β-glucan recognition by dectin-1 include the production of various cytokines, including TNF-α, IL-6, and IL-22, generation of reactive oxygen species (ROS), as well as expression of neutrophil chemoattractants, MIP-1α and MIP-2 (Hohl et al., 2005; Underhill et al., 2005; Gravelat et al., 2013). Collectively, previous observations suggest that recognition of β-glucan on the surface of germinating *Aspergillus* by dectin-1 is a critical early step in initiating anti-fungal immune responses (Hohl et al., 2005; Steele et al., 2005; Gersuk et al., 2006; Werner et al., 2009; Rivera et al., 2011).

Phagocytosis of a microbe such as fungal conidia initiates a process known as respiratory burst. Oxygen consumption by the phagocyte increases and the enzyme NADPH oxidase relocates to the phagosome and produces ROS to help contain and eliminate the invading threat (Nauseef, 2008). The importance of NADPH oxidase in host defense is best illustrated in patients suffering from chronic granulomatous disease (CGD). These individuals lack or have impaired NADPH function, and frequently contract severe bacterial and fungal infections (Ben-Ari et al., 2012). ROS can directly kill microbes by causing oxidative damage to their DNA, cell membrane, and wall components (Ramírez-Quijas et al., 2015; Nguyen et al., 2017), and indirectly contribute to clearance of hyphae by promoting the formation of neutrophil extracellular traps (NETs) (Bruns et al., 2010; Rohm et al., 2014; Jin et al., 2016). Defective generation of ROS in NADPH-deficient mice often results in exaggerated inflammation even after challenge with inactivated fungal cells (de Luca et al., 2014; Bagaitkar et al., 2015). In aggregate, these findings indicate that ROS are essential regulators of the host response to fungal infection both as direct effectors of fungal cell inactivation and regulators of inflammation.

Dectin-1 is known to stimulate ROS production in response to zymosan via the Syk pathway (Gantner et al., 2003; Underhill et al., 2005). In addition, a link between STAT1 activation and ROS generation has been previously reported (Yamashita et al., 2013; Kim et al., 2017; Wang et al., 2018), suggesting that IFNs may also contribute to this process. Indeed, our own findings established that both type I and type III IFNs have critical roles in potentiating ROS production by neutrophils in response to pulmonary infection with *Af* (Espinosa et al., 2017). However, the relationship between IFN-λ and dectin-1 is still unknown. In this study, we investigated whether recognition of *Af* β-glucan and signaling through dectin-1 impacts downstream production of type I and III IFNs. We find that dectin-1-dependent recognition of β-glucan exposure during *Af* germination induces increased production of type I and III interferons.

## Materials and Methods

### Experimental Set Up

C57BL/6 and dectin-1^−/−^ (Saijo et al., 2007) mice were bred in-house at the Rutgers New Jersey Cancer Center Research Animal Facility. All experiments were performed under BSL-2 guidelines and were approved by the Institutional Animal Care and Use Commitee at Rutgers University. Each study consists of at least two independent experiments each containing four to six mice per group. A BD LSR Fortessa X-20 was used for all flow cytometric analysis.

### *In vivo* Infections and Murine Models

Mice were challenged via non-invasive intratracheal delivery (Rivera et al., 2005) of either 6 × 10^7^ of resting-HK conidia, or 5 × 10^7^ of either live or swollen-HK conidia in a volume of 50 μl. Resting-HK conidia were generated by heat inactivation of spores immediately after collection. For swollen-HK prepartions, conidia were germinated in RPMI containing 10% FBS and 10% voriconazole for 18 h at 37°C before heat inactivation the following day. Mice were euthanized according to IACUC protocol at either 3 h post-infection (peak of IFN-α transcription) to harvest lungs, or at 48 h post-infection (peak of IFN-λ and ROS expression) to harvest lungs and BALs. Mice were exsanginuated and lungs were perfused with 10 ml of phosphate-buffered saline (PBS) to remove excess blood.

### Isolation of Murine Lungs

One lobe of each lung sample was homogenized by passage through a 70 μm strainer to use for protein analysis by ELISA. The remaining lung portions were minced and digested at 37°C in PBS with collagenase type IV (3 mg/ml, Worthington) to obtain single cell suspensions, then lysed to remove red blood cells. Lung suspensions were divided for analysis by flow cytometry and extraction of total lung RNA by Trizol. 48 h suspensions were also plated on saboraud agar plates by serial dilution for CFUs.

### Curdlan Treatment

Mice were treated intratracheally with 100 μg curdlan (Sigma) suspended in PBS. Lungs were harvested at 48 h for analysis.

### Measurement of ROS

Cells (1-2 × 10^6^) from BALs were incubated with 1 μM of CM-H2DCFDA (Life Technologies) in pre-warmed Hank's Balanaced Salt Solution at 37°C for 45–50 min. Afterward, cells were surface stained for 10 min and analyzed by flow cytometry.

### Histological Analysis

Survival experiments were terminated at either day 10 or 15 post-infection, and mice that developed severe symptoms of invasive aspergillosis beforehand (hunched posture, impaired breathing, difficulty, or lack of motion, unable to eat) were euthanized. Lungs were perfused in PBS and fixed in 10% formalin. Fixed lungs were paraffin embedded and stained with Gomori-methenamine silver stain by the Histology Core Facility at Rutgers NJMS. Stained tissue sections were imaged at 40x magnification using a Leica confocal microscope and Surveyor software.

### IFN Treatment

Wild-type and dectin-1^−/−^ mice were infected with live CEA10 conidia as outlined above. On the day of and 1 day following infection, a cohort of dectin-1^−/−^ were injected intraperitoneally with 1 μg each of IFN-α2 (Novoprotein) and pegylated-IFN-λ3 (PBL). Mice were then euthanized at 48 h and lungs and BALs assessed as described. For survival experiments, mice were given doses of both IFN-α2 and pegylated-IFN-λ3 on the day of infection, and then every other day up to the end point.

### Statistical Analysis

GraphPad Prism software was used to conduct all statistical analysis using non-parametric tests.

## Results

### β-Glucan Exposure Promotes Expression of Type III Interferon in Response to Pulmonary Infection With *Af*, a Phenotype That Is Dependent on the Presence of Dectin-1

**Figure 1:**
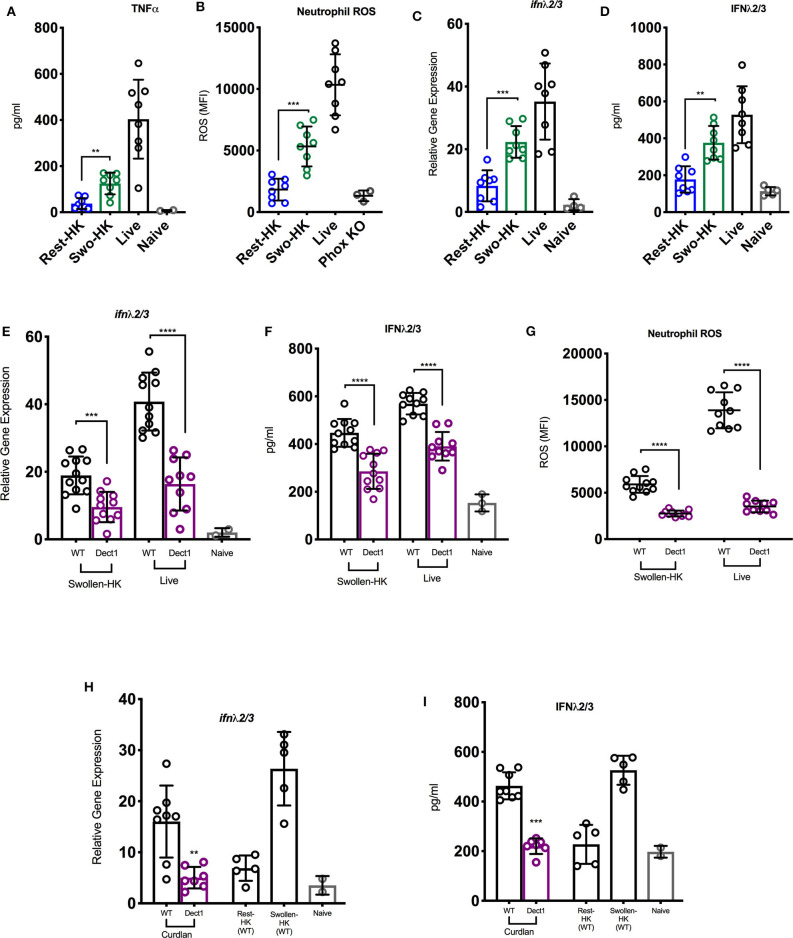
Exposure of β-glucan and sensing by dectin-1 promotes inflammatory responses to *A. fumigatus* and regulates optimal IFN-λ expression. **(A–D)** Wild-type C57BL/6J were challenged with 6 × 10^7^ resting-HK, 5 × 10^7^ swollen-HK, or 5 × 10^7^ live CEA10 conidia for 48 h and lung homogenates analyzed for production of **(A)** TNF-α, and **(D)** IFN-λ protein, and **(C)** mRNA expression of IFN-λ. **(B)** BAL fluids were analyzed for expression of neutrophil ROS in treated mice. BAL from _P47_Phox-/- mice challenged with live conidia was used as control for baseline staining for ROS. **(E–G)** Wild-type C57BL/6 (black bars and symbols) or dectin-1^−/−^ (purple bars and symbols) mice were infected either with 5 × 10^7^ live or 5 × 10^7^ swollen-HK CEA10 conidia for 48 h. **(E,F)** Expression of IFN-λ mRNA and protein 48 h after infection with either swollen-HK (left) or live (right) conidia. **(G)** Production of ROS by BAL neutrophils in response to swollen-HK (left) or live (right) conidia. **(H,I)** Expression of IFN-λ mRNA **(H)** and protein **(I)** in response to IT treatment with curdlan. Wild-type mice challenged with resting-HK and swollen-HK CEA10 included as controls. Data is cumulative of two independent experiments. Each symbol represents one mouse. **P < 0.01; ***P < 0.001; ****P < 0.0001, calculated by Mann-Whitney non-parametric test.

In order to investigate whether exposure of fungal PAMPs was involved in the activation of an IFN cascade during infection with *Af*, we challenged mice with three distinct inoculums: heat-inactivated resting conidia, swollen heat-killed (HK) conidia, or live spores. These preparations have been previously shown to vary in their levels of β-glucan exposure (Hohl et al., 2005). Heat-inactivated resting *Af* conidia have been shown to induce minimal immune activation due to masking of β-glucan under an immunologically-inert hydrophobic layer (Aimanianda et al., 2009). As the conidia germinate, they expose increasing amounts of β-glucan and become sensitive to dectin-1 mediated recognition (Hohl et al., 2005; Steele et al., 2005; Gersuk et al., 2006). Consistent with these earlier studies, mice challenged with resting-HK conidia showed minimal induction of TNF-α, while swollen-HK conidia induced a significant response (Figure 1A). As expected, infection with live conidia induced an even more robust response (Figure 1A). The activation of ROS production by neutrophils was minimal in response to challenge with HK resting conidia, while increased β-glucan exposure in swollen-HK conidia correlated with a significant increase in ROS production (Figure 1B). Intriguingly, induction of type III IFN (IFN-λ) transcription was significantly increased in mice challenged with swollen-HK conidia as compared to mice challenged with resting-HK-conidia (Figure 1C). Higher levels of IFN-λ protein were also detected in the lungs of mice challenged with swollen-HK conidia as compared to mice challenged with resting-HK conidia (Figure 1D). The expression of IFN-λ was further boosted by infection with live conidia (Figures 1C,D). These observations suggest that induction of IFN-λ during infection with *Af* is influenced by exposure of fungal PAMPs during germination.

Since β-glucan is the best characterized PAMP exposed during *Af* germination, we then investigated whether dectin-1-mediated recognition was involved in the induction of type III IFN production. To this end, we challenged dectin-1^−/−^ and control WT mice with swollen-HK or live spores and examined ROS production and IFN expression. Dectin-1^−/−^ mice showed reduced expression of IFN-λ after challenge with swollen-HK or live conidia (Figures 1E,F) as compared to the induction observed in control mice (Figures 1E,F). Reduced expression of IFN-λ in dectin-1^−/−^ mice was detected at both the RNA and protein levels (Figures 1E,F). Diminished IFN-λ expression in dectin-1^−/−^ mice was also accompanied by reduced production of ROS by neutrophils (Figure 1G). Furthermore, treatment with curdlan, a linear β-1,3-glucan, induced expression of IFN-λ in WT mice (Figures 1H,I). The response to curdlan was drastically reduced in the absence of dectin-1. Altogether, these observations suggest that sensing of β-glucan exposure by dectin-1 is required for the optimal induction of IFN-λ expression and activation of ROS in neutrophils.

### Dectin-1 Is Required for Control of Invasive Aspergillosis Upon Infection With *Af*-CEA10 Strain

**Figure 2:**
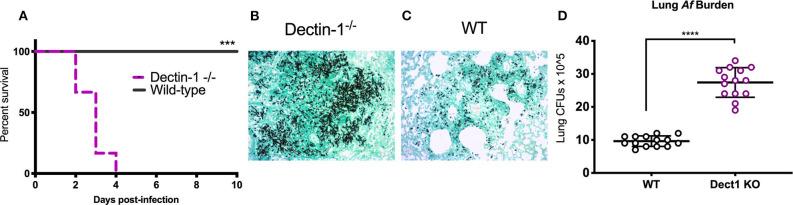
Dectin-1 signaling is required to control fungal infection and prevent invasive aspergillosis. Wild-type C57BL/6 or dectin-1^−/−^ mice were infected with 8 × 10^7^ live CEA10 conidia then monitored for survival for 10 days. **(A)** Kaplan-Meir survival plot for 10 mice per group analyzed in two independent experiments. **(B,C)** Histological samples showing representative GMS staining of dectin-1^−/−^ and wild-type mice lung sections. **(D)** Pulmonary fungal burden at 48 h after infection in dectin-1^−/−^ and control wild type mice. Data is cumulative of two independent experiments. For CFUs, each symbol represents one mouse. Each survival experiment was performed twice with five mice per group for a total of 10 mice per group. **(A)** ***P < 0.001 as determined by log-rank (Mantel-Cox) test. **(D)** ****P < 0.0001, calculated by Mann-Whitney non-parametric test.

In our previous work, we uncovered that IFN-λ is an essential regulator of antifungal neutrophils and required for optimal ROS production and prevention of IA in mice infected with *Af*-CEA10 (Espinosa et al., 2017). The data in Figure 1 further show that sensing of β-glucan by dectin-1 is linked to optimal production of IFN-λ and ROS. We thus hypothesized that dectin-1 signaling might be required for host defense against infection with *Af*-CEA10. Consistent with this idea, we observed that 100% of dectin-1^−/−^ infected with *Af*-CEA10 succumbed to infection in contrast to the long-term survival of WT control mice (Figure 2A). Importantly, mortality in dectin-1^−/−^ mice was linked to the development of invasive fungal growth in the lung (Figure 2B) as compared to the efficient containment of conidia seen in wild type (Figure 2C). Dectin-1^−/−^ mice showed increased fungal burden in the lung 48 h after infection as compared to control mice (Figure 2D), further suggesting a rapid loss of fungal growth control. Altogether, these observations indicate that dectin-1 signaling is required for defense against infection with the rapidly germinating isolate *Af*-CEA10.

### Dectin-1 Promotes the Induction of Type I IFN Expression

**Figure 3:**
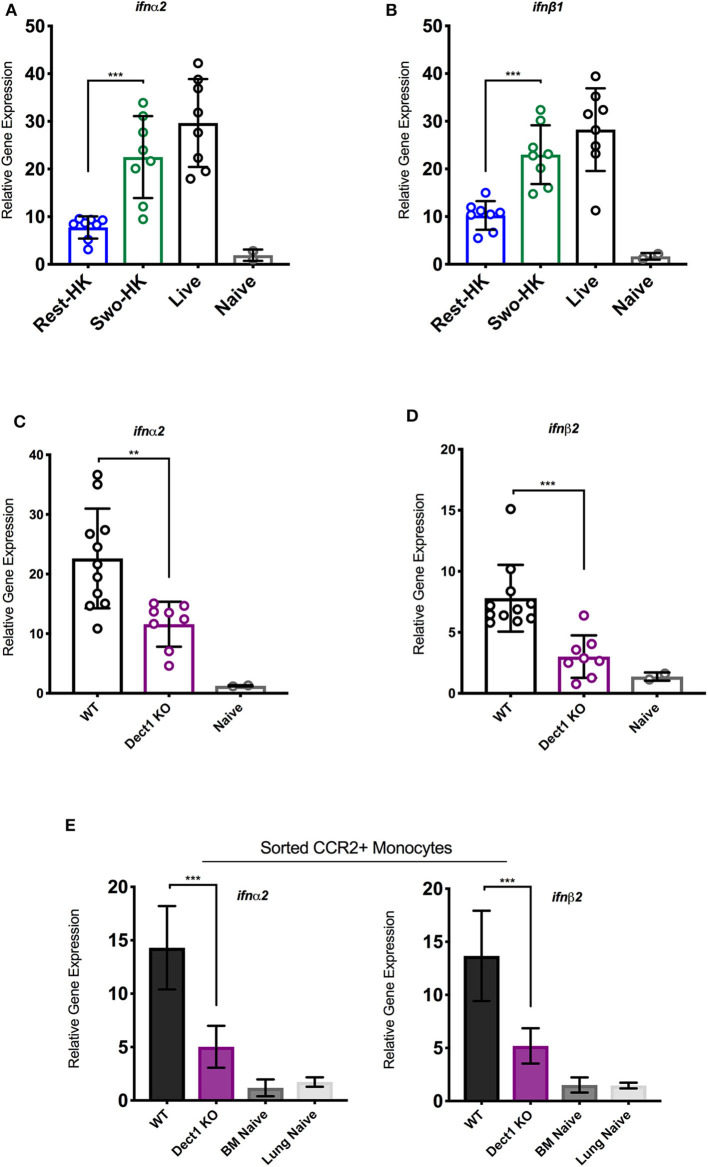
Dectin-1-mediated recognition of β-glucan promotes type I IFN expression by CCR2+ monocytes. To assess the contribution of β-glucan exposure to type I IFN expression, wild-type C57BL/6J were challenged with 6 × 10^7^ resting-HK, 5 × 10^7^ swollen-HK, or 5 × 10^7^ live CEA10 conidia for 3 h, at which point lungs were collected. **(A,B)** Total RNA from wild-type lung homogenates were analyzed for expression of IFN-α and IFN-β in response to different stages of spore germination by qRT-PCR. To assess the contribution of dectin-1 signaling, wild-type C57BL/6 or dectin-1^−/−^ mice were infected with 5 × 10^7^ swollen-HK CEA10 conidia for 3 h and lungs were collected. **(C,D)** Comparison of IFN-α and IFN-β gene expression by qRT-PCR. Data is cumulative of two independent experiments. Each symbol represents one mouse. Inflammatory monocytes (DAPI^−^CD45^+^CD11b^+^NK1.1^−^SiglecF^−^ CD11c^−^Ly6C^+^Ly6G^−^CCR2^+^) were sorted by flow cytometry from lungs of wild-type and dectin-1^−/−^ mice infected with CEA10 for 3 h, and lungs, and bone marrow (BM) of naïve wild-type mice. **(E)** Eight mice were pooled for each group and gene expression of IFN-α and IFN-β assessed in quadruplicate, two independent times. Data shown is for mean ± SEM relative gene expression normalized to GAPDH from two independent experiments. **P < 0.01, ***P < 0.001 as determined by Mann-Whitney non-parametric test.

In our previous studies, we uncovered that optimal expression of IFN-λ upon infection with *Af* is critically shaped by the rapid production of type I IFNs (Espinosa et al., 2017). In the context of *Candida* infection, dectin-1 has been shown to promote type I IFN expression (del Fresno et al., 2013). Therefore, we hypothesized that dectin-1-dependent recognition of β-glucan shapes the IFN-λ response by also affecting the initial induction of type I IFN. To test this hypothesis, we examined the early production of type I IFN in mice challenged with heat-killed resting and swollen conidia as compared to mice infected with live spores. At 3 h after infection, mice challenged with HK-swollen conidia had significantly higher expression of IFN-α2 and IFN-β1 as compared to mice challenged with HK-resting conidia (Figures 3A,B). As expected, mice infected with live conidia showed even higher expression of type I IFNs (Figures 3A,B). Dectin-1^−/−^ mice showed significantly reduced expression of type I IFNs as compared to wild type control mice (Figures 3C,D). These data suggest that early sensing of β-glucan exposure by dectin-1 helps initiate the type I IFN response. Since our earlier work identified CCR2^+^ monocytes as a critical source of early type I IFN (Espinosa et al., 2017), we examined the impact of dectin-1 deficiency on these cells. We observed that sorted CCR2^+^ monocytes obtained from dectin-1^−/−^ mice expressed significantly less type I IFNs as compared to monocytes isolated from control mice (Figure 3E). In aggregate, our observations indicate that dectin-1 signals are crucial for the activation of an optimal type I and III IFN response upon infection with the virulent *Af*-CEA10 strain.

### Exogenous Administration of IFNs Promotes Improved Fungal Growth Control in Dectin-1^-/-^ Mice

**Figure 4:**
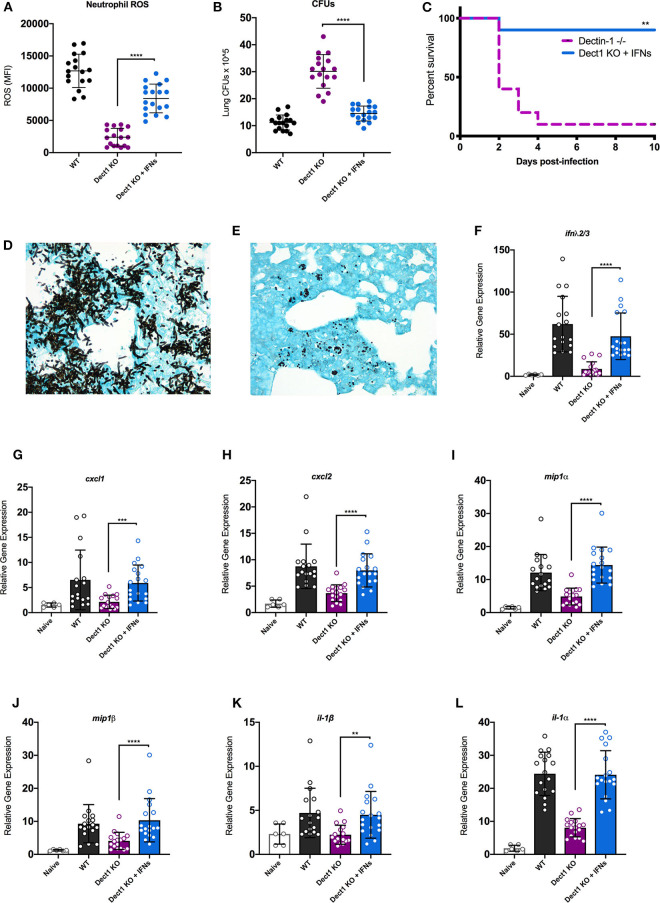
Administration of recombinant type I and III IFNs promotes enhanced antifungal responses in dectin-1^−/−^ mice. Wild-type C57BL/6 or dectin-1^−/−^ mice were infected with 5 × 10^7^ live CEA10, and a group of dectin-1^−/−^ mice treated with IFN-α and IFN-λ on the day of and 1 day following infection. **(A)** Neutrophils from bronchial lavage fluid were assessed for generation of ROS. **(B)** Lung suspensions were plated by serial dilution for assessment of fungal burden at 48 h. **(C)** Survival of dectin-1^−/−^ mice treated with type I and III IFNs, in comparison to untreated mice. **(D,E)** Representative images of GMS staining of lung tissue from untreated **(D)** and IFN treated **(E)** dectin-1^−/−^ mice. Each group was infected with 8 × 10^7^ live CEA10 conidia. **(F–L)** Total lung RNA was assessed for expression of IFN-λ **(F)**, CXCL1 **(G)**, CXCL2 **(H)**, MIP1α **(I)**, MIP1β **(J)**, IL1β **(K)**, and IL1α **(L)**. Gene expression was examined by qRT-PCR with TaqMan probes and calculated as relative gene expression normalized to GAPDH housekeeping gene. Data shown is cumulative of four independent experiments. Each symbol represents one mouse. **P < 0.01, ***P < 0.001, ****P < 0.0001 as determined by Mann-Whitney non-parametric test.

In our previous work, we uncovered that type I and III IFNs are necessary for the activation of antifungal neutrophils (Espinosa et al., 2017). We further showed that optimal defense against *Af* was orchestrated by the coordinated actions of both type I and III IFNs (Espinosa et al., 2017). We thus hypothesized that impaired activation of antifungal responses in dectin-1^−/−^ mice (Figure 2) might be, at least in part, due to the defective induction of IFNs. To test this hypothesis, we treated dectin-1^−/−^ with recombinant type I and III IFNs and tested the impact of this treatment to ROS production by neutrophils and global control of CFU in the lung. Administration of IFNs significantly restored the production of ROS by neutrophils (Figure 4A). Moreover, improved generation of ROS by neutrophils was accompanied by significantly enhanced control of fungal growth in the lung (Figure 4B). We also observed that administration of recombinant type I and III IFNs to dectin-1^−/−^ mice resulted in significantly improved resistance to *Af* infection (Figure 4C). These data provide support for the idea that impaired antifungal immunity in dectin-1^−/−^ mice is linked to defective type I and III IFN expression. Importantly, administration of recombinant type I and III IFNs can help restore containment of hyphal growth in mice with defective dectin-1 (Figures 4D,E), further supporting the potential use of IFNs as a novel immune-modulating therapy to boost antifungal immunity.

Previous studies have documented that dectin-1-dependent signals promote the expression of multiple cytokines and chemokines (Vautier et al., 2012). We thus hypothesized that the restoration of fungal growth control in interferon-treated dectin-1^−/−^ mice might be linked, at least in part, to activation of genes previously reported as being affected in dectin-1^−/−^ mice. To test this hypothesis, we examined the impact of IFN treatment to the expression of a selected set of genes previously shown to be defective in dectin-1^−/−^ infected with *Af* (Werner et al., 2009). We also observed that IFN treatment boosted the expression of endogenous IFN-λ, consistent with our previous work (Figure 4F). Furthermore, IFN treatment helped improve the expression of all the tested genes as compared to untreated dectin-1^−/−^ mice (Figures 4G–L). A particularly striking improvement in expression was seen for IL-1α (Figure 4L), a previously identified important regulator of defense in mice infected with *Af*-CEA10 strain (Caffrey et al., 2015; Caffrey-Carr et al., 2017). Altogether, our findings support the conclusion that dectin-1-dependent recognition of β-glucan in germinating *Af* conidia helps promote optimal production of type I and III IFNs. In turn, type I and III IFNs are critical activators of some of the previously associated, dectin-1-dependent antifungal responses.

## Discussion

In this study, we uncovered that the optimal production of type I and III IFNs in response to pulmonary infection with *Af* requires intact dectin-1 signaling. Importantly, we further show that defective innate responses in dectin-1^−/−^ mice can be significantly improved by exogenous administration of type I and III IFNs. Our findings suggest a link between fungal germination, exposure of β-glucans, and dectin-1 engagement to the activation of an interferon-dependent cascade of antifungal responses. Thus, an important role for dectin-1 signals in antifungal immunity appears to be linked to the induction of type I and III interferons. Consistent with this idea, exogenous administration of IFNs in dectin-1^−/−^ mice was able to improve expression of inflammatory mediators, ROS generation by neutrophils and overall control of fungal burden. These findings support the further exploration of type I and III IFNs as potential immune based therapies to improve antifungal dysfunctions in various clinically relevant settings.

Previous studies have examined the role of dectin-1 in overall defense against IA and found varying degrees of susceptibility depending on the fungal strain used. Dectin1^−/−^ mice infected with Af293 showed minimal defects in host immunity and were resistant to IA (Jhingran et al., 2012). In contrast, infection with isolate 13073 led to increased mortality and defective induction of inflammatory cytokines (Werner et al., 2009). In the current study, we determined that defense against infection with *Af*-CEA10 required intact dectin-1 signals. This observation is consistent with studies in *Candida albicans*, where differential roles for dectin-1 were also reported for various strains (Marakalala et al., 2013). The observations of our study together with previous work suggest that *Af* strains that rapidly germinate and expose β-glucans differentially engage ensuing host immune responses and are more virulent in hosts with innate defects. The findings in our study suggest that rapid recognition of β-glucan by dectin-1 helps activate a protective interferon response that culminates in the optimal activation of antifungal neutrophils.

Our recent work documented for the first time a requirement for type I and III interferons for the activation of antifungal neutrophils. Previous studies on these cytokines were primarily focused on their role as potent effectors of defense against viruses. In this context, the primary innate sensing pathways described so far to promote type I and III interferon production are those that detect the presence of viral-derived nucleic acids. The detection of nucleic acids by members of the TLR family (TLRs 3,7, and 9) and/or by cytosolic sensors STING, RIG-I, and downstream MAVS are potent activators of type I and III interferon expression (Levy et al., 2011). The activation of type III interferon expression upon infection with *Af* that we previously uncovered was unexpected and led us to investigate whether fungal-derived molecular patterns may be involved in the regulation of interferon expression. Indeed, in the current study we document that recognition of β-glucan by dectin-1 during the process of fungal germination helps activate the type I and III IFN cascade.

Another important implication of our study is the potentially expanded utility of using exogenous administration of type I and III interferons as immunotherapies to combat fungal infections. In our previous work, we showed that combination treatment with type I (IFN-α2) and type III (IFN-λ3) IFNs was able to enhance the function of neutrophils in monocytopenic mice (Espinosa et al., 2017). In the current study, we found that administration of the same combination of IFNs to dectin-1^−/−^ mice helped improve neutrophil function and overall control of fungal infection in the lung. Ongoing efforts in our lab are focused in testing the potential benefit of combination IFN therapies in diverse mouse models of susceptibility to IA. Recombinant type I IFNs, including pegylated IFN-α2, are FDA-approved therapeutics in current use against a variety of conditions including various malignancies and viral infections. Formulations of pegylated-IFN-λ are currently in phase 2 trials and have shown good promise for patient tolerability of side effects and efficacy against hepatitis B infection (Phillips et al., 2017). Therefore, future studies from our lab and others could provide critical proof-of-principle data to support future clinical trials to test the potential use of combination treatments with type I and III IFNs in the improvement of immune function and antifungal defense in susceptible patients.

## Data Availability Statement

All datasets generated for this study are included in the article/supplementary material.

## Ethics Statement

The animal study was reviewed and approved by Rutgers University Institutional Animal Care and Use Committee.

## Author Contributions

AR and OD conceived the project, designed the experiments, analyzed the data, and made figures and co-wrote the manuscript. OD, VE, KW, and SA performed experiments and analyzed data. All authors contributed to the article and approved the submitted version.

## Conflict of Interest

The authors declare that the research was conducted in the absence of any commercial or financial relationships that could be construed as a potential conflict of interest.
